# Viable *Clostridium botulinum* spores not detected in the household dust of major Canadian cities

**DOI:** 10.1017/S0950268823001474

**Published:** 2023-08-07

**Authors:** Richard A. Harris, Madeleine Blondin-Brosseau, Christine Levesque, Pat E. Rasmussen, Suzanne Beauchemin, John W. Austin

**Affiliations:** 1 Bureau of Microbial Hazards, Health Canada, Ottawa, ON, Canada; 2 Environmental Health Science and Research Bureau, Health Canada, Ottawa, ON, Canada

**Keywords:** anaerobic bacteria, *Clostridium botulinum*, epidemiology, paediatrics, infant botulism

## Abstract

*Clostridium botulinum* causes infant botulism by colonising the intestines and producing botulinum neurotoxin *in situ.* Previous reports have linked infant botulism cases to *C. botulinum* spores in household dust, yet the baseline incidence of *C. botulinum* spores in residential households is currently unknown. Vacuum cleaner dust from 963 households in 13 major Canadian cities was tested for *C. botulinum* using a novel real-time PCR assay directed against all known subtypes of the botulinum neurotoxin gene. None of the samples tested positive for *C. botulinum.* Analysis of a random subset of samples by MALDI Biotyper revealed that the most common anaerobic bacterial isolates were of the genus *Clostridium* and the most common species recovered overall was *Clostridium perfringens.* Dust that was spiked with *C. botulinum* spores of each toxin type successfully produced positive real-time PCR reactions. These control experiments indicate that this is a viable method for the detection of *C. botulinum* spores in household dust. We make several recommendations for future work that may help discover a common environmental source of *C. botulinum* spores that could lead to effective preventative measures for this rare but deadly childhood disease.

## Introduction

*Clostridium* is a genus of Gram-positive spore-forming anaerobic bacteria that are widely found in terrestrial soils, aquatic sediments, and human gastrointestinal tracts. *Clostridium botulinum*, and rare strains of *Clostridium butyricum* and *Clostridium baratii*, produce botulinum neurotoxin (BoNT), which is the primary causative factor in the paralytic disease botulism. There are five main groups of BoNT-producing clostridia, of which group I (proteolytic) and group II (non-proteolytic) are most commonly associated with human disease and produce BoNT serotypes A, B, and F, and B, E, and F, respectively [1]. Infant botulism is a rare form of intestinal toxemia (colonisation) botulism that occurs in children less than one year of age and is now the most common form of botulism in Canada (58% of cases from 2015 to 2019) [2].

In contrast to foodborne botulism, in which a common food source can be identified by linkage to multiple cases during an outbreak, the ubiquity of *C. botulinum* spores in the environment combined with the sporadic nature of infant botulism makes source attribution a significant challenge. From 1979 to 2019, 95% of all infant botulism cases in Canada did not have a laboratory-confirmed source [2]. Of the 5% that were identified, all were attributed to ingestion of contaminated honey, a well-established and commonly known source of spores [3, 4]. Evidence indicates that household dust could also be a source of *C. botulinum* infection. In 2002 and 2010, isolates of *C. botulinum* from infant botulism patients’ intestinal contents were matched to isolates obtained from vacuum cleaner dust and household dust, respectively [5, 6].

Household dust is comprised of a well-documented microbiome that generally matches the commensal bacteria of the inhabitants [7]. The baseline incidence of *C. botulinum* spores in residential households is currently unknown. A small-scale study from California in 1981 found that approximately 10% of household dust samples contained *C. botulinum* isolates [8]. Previous studies have successfully recovered *C. botulinum* spores from outdoor soil and indoor dust from households associated with infant botulism cases that often matched the BoNT serotype with clinical isolates [8–14]. However, recent attempts to identify a wide range of environmental risk factors for infant botulism in California, including exposure to dust, hygiene practices, food history, and pet ownership, were largely inconclusive [15, 16].

In this study, we investigated 963 household vacuum cleaner dust samples for the presence of viable *C. botulinum* spores using a novel real-time PCR assay directed against all known subtypes of *bont* genes A, B, E, and F. We also used the MALDI Biotyper to identify common anaerobic bacterial species isolated from a random subset of household dust samples.

## Methods

### Dust sample processing

A set of 963 vacuum cleaner dust samples was obtained from the Canadian House Dust Study (CHDS) [17]. Briefly, the CHDS was designed to provide nationally representative house dust samples for typical urban Canadian homes. Two types of settled dust (householder vacuum cleaner bags and professionally sampled composite fresh vacuum dust) were collected in the winter seasons from January 2007 to March 2010 from houses in 13 major cities across Canada with a population over 100,000 [17]. Dust samples were air-dried, homogenised (large particles manually removed), sieved into fine (<80 μm) and coarse (80–300 μm) size fractions, and stored in amber glass bottles at room temperature. For this study, dust samples from household vacuum bags were used to ensure sufficient sample mass for all homes. Previous comparisons of CHDS sampling approaches demonstrated the advantage of using vacuum cleaner dust as a representative, cost-effective sample of the whole house with a longer accumulation time compared with the brief two-hour sampling of fresh dust [18, 19]. Coarse fraction (80–300 μm) dust samples were weighed out to 150 mg ± 50 mg using an analytical balance (Sartorius, ED124S) into 5-mL sterile polystyrene culture tubes (BD Falcon) in a biological safety cabinet.

### Culture conditions

Dust samples were inoculated with 3 mL of deaerated TPGY (5% tryptone (BD), 0.5% peptone (Oxoid), 0.4% glucose (BD), 2% yeast extract (BD), and 0.1% sodium thioglycolate (Sigma-Aldrich)) and anaerobically incubated at 35 °C for 24 hours and then at 30 °C for 24 hours using an anaerobic workstation (Baker Ruskinn, Concept 400). Cultures were briefly vortexed, and then, 200 μL was aliquoted to 2 mL screw cap centrifuge tubes and spun at 21,000 rcf for 5 minutes at room temperature. The pellet was resuspended in 100 μL of certified nuclease-free TE (Tris-EDTA, pH 8.0) buffer (IDT) and boiled for 10 minutes. The tube was centrifuged again at the previous settings to settle the condensation, and 1 μL of supernatant was used as a template for the real-time PCR assay.

### Real-time PCR

Multiplex real-time PCR was used for the detection of *C. botulinum* using newly designed primers and probes with 100% alignment to all currently known subtypes of *bont* genes A, B, E, and F without the use of degenerate bases (Table 1). Two separate sets of multiplex reactions were developed: one for the detection of *bont* A subtypes 1–8, B subtypes 1–8, and E subtypes 1–12; and a separate multiplex reaction for the detection of *bont* F subtypes 1–4, 6, 8–9, F subtype 5, and F subtype 7. Positive controls were used for all reactions using purified DNA from *C. botulinum* strains (Table 2) and gBlock gene fragments (IDT). A common internal amplification control (IAC) was used in all reactions using a linearised custom pUC plasmid (Blue Heron) at a final concentration of 10 fg/μL (Table 3). All reactions were prepared in an AC600 PCR Workstation (AirClean) using Luna Universal Probe qPCR Master Mix (NEB) and performed in duplicate in a calibrated CFX96 Thermal Cycler (Bio-Rad) according to the following parameters: 10 minutes at 95 °C, then 40 cycles of 15 seconds at 95 °C and 20 seconds at 58 °C, and then hold at 10 °C. A positive result was identified as an exponential increase in fluorescence with a defined Ct value below 35 and an end signal above 100 relative fluorescence units (RFUs). This method was previously validated for specificity and sensitivity according to ISO 17025:2017 standards and is currently in use for the detection of *bont* genes A, B, E, and F in food and stool with a 100% agreement with the mouse bioassay for the past 59 investigations since 2021 including 15 positive samples.

**Table 1.** Primer and probe sequences and reporters/quenchers

Primer or probe	Sequence (5′-3′)	Reporter/quencher
BoNT A-Fwd	CTACTCAATACATTAGATTTAGCCC	N/A
BoNT A-Rev	GCCTGCACCTAAAAGAGG	N/A
BoNT A-Probe	TTGGTTTTGAGGAGTCACTTGAAGTTGA	FAM/BHQ1
BoNT B-Fwd	TGGGTGAAAAGTTATTAGAGATG	N/A
BoNT B-Rev	GGCCCAGGTCCAAATATTAT	N/A
BoNT B-Probe	TCTTCGAGTGGAACACGTCTATCTCC	Cy5/BHQ2
BoNT E-Fwd	CCAAAATATGATTCTAATGGAACAAG	N/A
BoNT E-Rev	ATATCTGCAATTTTATCAACAGTAC	N/A
BoNT E-Probe	CACAGAAAGTGCCCGAAGGTGA	HEX/BHQ1
BoNT F-Fwd	CTAATTATTTAACCACTGATGCTG	N/A
BoNT F-Rev	GCAGGATCTGCAATAAATGATT	N/A
BoNT F-Probe	AATCTTCTTGTATTGGGAGCAGGACCT	FAM/BHQ1
BoNT F5-Fwd	ATGAAGCCGAAGTTGGTAAAT	N/A
BoNT F5-Rev	GGTCTGTAGTGTTTAGGAATCC	N/A
BoNT F5-Probe	TCTTAGGAGAGCAGGGAGGGGAAT	FAM/BHQ1
BoNT F7-Fwd	GGTTTATACGGAGCTAAAGGTGT	N/A
BoNT F7-Rev	CAGTATAGTTCCCATTAGAATCTTG	N/A
BoNT F7-Probe	AAGTCATAGAAGTAGATCAGGGTGCCC	FAM/BHQ1
IAC-Fwd	AGGAGTAAGTGCATTGAGG	N/A
IAC-Rev	TGCCAATAATACGACGCTTA	N/A
IAC-Probe	TCTTGCTTGAGGATCTGTCGTGGA	Cy5.5/BHQ3

**Table 2.** Positive control DNA source and expected amplicon sizes

Target	DNA source (strain)	Stock Conc. (pg/μL)	Amplicon size (bp)
A	Hall A	100	89
B	15044	100	184
E	FWKR11E1	100	302
F	Langeland	100	457
F5	gBlock F5	1	463
F7	gBlock F7	1	304
IAC	Linearised pUC (plasmid)	0.01	289

**Table 3.** Sequence of internal amplification control (IAC) in pUC cloning plasmid

Cloning plasmid	Insert	Sequence (5′-3′)
pUC	Internal amplification control (IAC)	CGGCTCTAGCGCTGGTGGAGGTTAGAGTTCTCTGACATACGTGCTTCTGAACGGTAGGGAGTTGACGGACTGAGGGTAGGAGTGCTTAGCGTAGGAGTATTAGGTGGCGTGCTGTGGTGGTCGCGTGTGGGTTTAAGGAGTAAGTGCATTGAGGATTCAGGTGACACAGTAACGTGCAGTTGACTGGTGATGCCGCATTATTACTAGGCGATTCTTGCTTGAGGATCTGTCGTGGATCGGGGAGCGCAAACCTTACATGATATGTCTAAAATAGCTTTATGCCCTGCGATCGACCATATTTAAAAGCCTGCGACTGCTAATACCTATAGACTGAGGAGGGATTGAGAGAGCGAAAAATAGAGCAGACTGTATGATTACTATCGCGTGCCATCTCTAACTTTGCATAAGCGTCGTATTATTGGCAGCTACGAGTATCACGATTAGTCCGAACTAGTGGC

### MALDI Biotyper

A subset of three randomly chosen dust samples from each of the 13 major cities (39 total) was weighed out and cultured as previously described. After incubation, 10 μL of culture was streaked onto MT-EYE agar plates (McClung Toabe agar (HiMedia) mixed with egg yolk emulsion prepared fresh from homogenising two egg yolks into 100 mL of sterile saline). Plates were incubated at 35 °C for 24 hours and then at 30 °C for 24 hours using an anaerobic workstation (Baker Ruskinn, Concept 400). Two colonies of each unique morphology were selected from each plate and identified using the MALDI Biotyper IVD system based on Microflex LT (Bruker Daltonics). Briefly, bacterial colonies were resuspended in 20 μL of sterile water and then 2 μL was applied as a thin film onto a 96-spot steel plate (Bruker Daltonics) and allowed to dry at room temperature. Spots were overlayed with 2 μL of MALDI matrix: a saturated solution of α-cyano-4-hydroxycinnamic acid (Bruker Daltonics) in standard organic solvent (50% acetonitrile and 2.5% trifluoroacetic acid) (Sigma). Spectra were analysed by the MBT Compass v4.1 software (Bruker Daltonics) using the BDAL library v11 (11,897 entries), in addition to a custom library of 324 strains of *C. botulinum*, and a bacterial test standard (Bruker Daltonics) for calibration. The identification score criteria used were recommended by the manufacturer: a score of >2.0 indicated high-confidence identification (species level), a score of 1.7–2.0 indicated low-confidence identification (genus level), and a score < 1.7 failed to identify the organism.

## Results

None of the cultures set up from the 963 dust samples tested positive for *bont* genes A, B, E, or F. Positive control DNA samples produced successful signals, as did the IAC for each individual reaction, and negative controls (TE only) produced no signals, collectively indicating successful runs. These results suggest that the incidence of *C. botulinum* spores in the households of major Canadian cities is less than 1 in 963.

To determine which anaerobic bacterial species were present in the dust samples, and to confirm that they were able to grow under these conditions, a subset of three samples for each of the 13 cities (39 total) was randomly selected and streaked onto MT-EYE agar plates for colony identification by MALDI Biotyper (Supplementary material S1). Of the 39 samples, 38 (97%) produced viable colonies. *Clostridium* was the most common (54%) genus identified (Table 4), and *Clostridium perfringens* was the most common (33%) species identified overall (Table 5).

**Table 4.** Genus of organisms unique to each dust sample identified by MALDI Biotyper (*n* = 54)

Genus	Count	Percent
*Clostridium*	29	54
*Enterococcus*	9	17
*Niallia*	4	7
*Bacillus*	4	7
*Paenibacillus*	3	6
*Cronobacter*	1	2
*Erwinia*	1	2
*Mixta*	1	2
*Pantoea*	1	2
*Paraclostridium*	1	2

**Table 5.** Species of organisms unique to each dust sample identified by MALDI Biotyper (*n* = 52)

Species	Count	Percent
*Clostridium perfringens*	17	33
*Clostridium tertium*	8	15
*Enterococcus faecium*	5	10
*Niallia circulans*	4	8
*Paenibacillus polymyxa*	3	6
*Enterococcus casseliflavus*	3	6
*Bacillus cereus*	3	6
*Clostridium budayi*	2	4
*Bacillus thermoamylovorans*	1	2
*Bacillus thuringiensis*	1	2
*Clostridium nitritogenes*	1	2
*Clostridium paraputrificum*	1	2
*Enterococcus mundtii*	1	2
*Mixta calida*	1	2
*Paraclostridium bifermentans*	1	2

To demonstrate that viable *C. botulinum* spores could be identified using this novel real-time PCR assay, a dust sample from the subset above (that was found to contain viable *C. perfringens* as a potential competitor) was inoculated with 1,000 spores/mg of *C. botulinum* for each strain of BoNT type A (62A), B (CDC7827), E (Dolman VH), and F (Langeland) and cultured and processed as previously described. This real-time PCR assay produced a positive reaction for cultures spiked with each of the strains (Supplementary material S2). Positive control DNA produced successful signals, as did the IAC for each reaction, and negative controls (TE only) produced no signal. These results demonstrate that the assay is a viable method for the detection of *C. botulinum* spores in household dust for each BoNT type.

## Discussion

Considering that resident clostridial spores in dust were capable of germinating under these culture conditions, and together with the successful real-time PCR detection of dust cultures spiked with *C. botulinum* spores, we suspect that false negatives in the samples tested for this study are unlikely. The germination of other clostridial spores supports the relevance of using the 80–300 μm dust fraction, despite the small size (4–6 μm × 0.9-1.2 μm) of *C. botulinum* spore rods [20]. *C. botulinum* spores are known to adhere to organic and inorganic surfaces including soil minerals, plants and invertebrates, and synthetic materials such as polypropylene [21–23]. Hydrophobic bonds and electrostatic forces play an active role in their adhesion to surfaces, and drying can further increase their adhesion [22–24]. They can survive for decades and are resistant to harsh environmental conditions [21, 23, 25]. Therefore, the air-dried, archived 80–300 μm dust fraction was a relevant substrate for this study. Preliminary tests on four control samples compared the <80 μm versus 80–300 μm dust fractions and did not detect positive results in the fine fraction (data not shown). It is possible, however, that subsampling a small mass of dust (150 mg) from a coarser dust fraction might have resulted in missed spores in the case of low-occurrence species.

Isolation of *C. botulinum* spores from indoor dust and outdoor soils associated with cases of infant botulism has been previously described [8–14]. One possible reason for the absence of isolates recovered from this study is a decreased exposure to airborne dust in urban households compared with cultivated rural areas. In 1985, a discrete geographic ring of infant botulism ‘hot spots’ was described in rural and suburban areas surrounding the city of Philadelphia, accounting for 83% of cases in counties that represented approximately 30% of the population in the state [10]. In 1989, a two-year prospective case-controlled study identified that the only significant risk factor for infants less than two months of age was living in a rural area or on a farm [26]. In 2007, a study in Argentina found that the highly populated central region, which includes Buenos Aires, accounted for a relatively high prevalence of spores in the soil yet a low incidence of infant botulism compared with the rural West and South regions [27]. However, additional environmental factors and case reporting could influence this association. The rural Northwest region of Argentina had a high prevalence of spores in the soil, and a low incidence of infant botulism was reported [27]. A recent descriptive epidemiological study of infant botulism in California from 1976 to 2016 found that the dense urban centres of San Francisco, Sacramento, Los Angeles, and San Diego had the highest incidence rates in the state of California (per 100,000 live births) [16]. In general, the distribution of infant botulism cases in California matched the underlying population, yet cases also occurred in less populated suburban and rural areas [15]. Future sampling strategies that include rural or suburban areas outside of major cities may yield more success in isolating *C. botulinum* spores from the environment.

Another explanation for the absence of *C. botulinum* spores in this study is the fact that infant botulism is less common in Canada (0.4 per 100,000 live births) than in the United States (2.0 per 100,000 live births), and the incidence may simply reflect the geographic distribution of spores in the surrounding environment [2, 28]. Soil samples from the state of California were found to harbour *C. botulinum* spores at the highest rate in the United States, with a serotype proportion of approximately 74% type A and 26% type B from typed cultures [29]. In recent years, cases of infant botulism in California occurred at an incidence threefold higher than in the rest of the country and with a closely reflected serotype proportion of 62% type A and 38% type B [15]. In Canada, the western coastal sediments of British Columbia are associated with *C. botulinum* type A and E spores [1]. Infant botulism cases in the western provinces of Alberta and British Columbia were exclusively associated with group I *C. botulinum* type A (type E belongs to group II) [2]. Therefore, individual cases of infant botulism could be an indication of a higher incidence of *C. botulinum* spores in the surrounding environment, and robust sampling of indoor dust and outdoor soils in the local areas associated with previous cases of infant botulism may be a better strategy to detect the presence of *C. botulinum.* In addition, thoroughly investigating all possible food and environmental samples from each instance of laboratory-confirmed infant botulism would help to determine the origin of infection on a case-by-case basis. These future endeavours may reveal a common environmental source of *C. botulinum* that leads to effective preventative measures for this rare but deadly childhood disease.

## Supporting information

Harris et al. supplementary material 1 Harris et al. supplementary material

Harris et al. supplementary material 2 Harris et al. supplementary material

## Data Availability

All relevant data are submitted as tables and supplementary files.

## Supplementary Material

The supplementary material for this article can be found at http://doi.org/10.1017/S0950268823001474.
